# The Association Between Route of Post-menopausal Estrogen Administration and Blood Pressure and Arterial Stiffness in Community-Dwelling Women

**DOI:** 10.3389/fcvm.2022.913609

**Published:** 2022-06-10

**Authors:** Cindy Z. Kalenga, Jacqueline L. Hay, Kevin F. Boreskie, Todd A. Duhamel, Jennifer M. MacRae, Amy Metcalfe, Kara A. Nerenberg, Magali Robert, Sofia B. Ahmed

**Affiliations:** ^1^Cumming School of Medicine, University of Calgary, Calgary, AB, Canada; ^2^Libin Cardiovascular Institute, University of Calgary, Calgary, AB, Canada; ^3^Faculty of Kinesiology and Recreation Management, University of Manitoba, Winnipeg, MB, Canada; ^4^Institute of Cardiovascular Sciences, St. Boniface Hospital Albrechtsen Research Centre, Winnipeg, MB, Canada; ^5^Alberta Kidney Disease Network, Calgary, AB, Canada; ^6^Alberta Children's Hospital Research Institute, University of Calgary, Calgary, AB, Canada; ^7^O'Brien Institute for Public Health, University of Calgary, Calgary, AB, Canada

**Keywords:** hormone therapy (HT), estrogen, blood pressure, arterial stiffness, augmentation index, pulse wave velocity, menopause, women

## Abstract

**Background:**

Postmenopausal hormone therapy (HT) is associated with increased cardiovascular risk. Although the route of estrogen administration may play a role in mediating risk, previous studies have not controlled for concomitant progestin use.

**Objective:**

To investigate the association between the route of estrogen therapy (oral or non-oral) HT use, without concomitant progestin, and blood pressure and arterial stiffness in postmenopausal women.

**Methods:**

Systolic blood pressure [SBP], diastolic blood pressure [DBP]), arterial stiffness (aortic pulse wave velocity [aPWV] and augmentation index at 75 beats per minute [AIx]) were measured using a validated automated brachial cuff-based oscillometric approach (Mobil-O-Graph) in a community-dwelling sample of 328 women.

**Results:**

Fifty-five participants (16.8%) were ever users (current and past use) of estrogen-only HT (oral [*n* = 16], transdermal [*n* = 20], vaginal [*n* = 19]), and 223 were never HT users (control). Ever use of oral estrogen was associated with increased SBP and DBP (Oral: SBP: 137 ± 4 mmHg, DBP: 79 ± 2 mmHg) compared to use of non-oral estrogen (transdermal: SBP: 118 ± 2 mmHg, DBP: 73 ± 1 mmHg; *p* < 0.01 & *p* = 0.012, respectively; vaginal: SBP: 123 ± 2 mmHg DBP: 73 ± 2 mmHg; *p* = 0.02 & *p* = 0.01, respectively.) and controls (SBP: 124 ± 1 mmHg, DBP: 74 ± 1 mmHg, *p* = 0.03, *p* = 0.02, respectively) after adjustment for covariates. aPWV was higher in oral estrogen ever users (9.9 ± 1 m/s) compared to non-oral estrogen (transdermal: 8.6 ± 0.3 m/s, *p* < 0.01; vaginal: 8.8 ± 0.7 m/s, *p* = 0.03) and controls (8.9 ± 0.5 m/s, *p* = 0.03) but these associations were no longer significant after adjustment for covariates. AIx was higher in oral estrogen (29 ± 2 %) compared to non-oral estrogen (transdermal: 16 ± 2 %; vaginal: 22 ± 1.7 %) but this association was no longer significant after adjustment for covariates (*p* = 0.92 vs. non-oral; *p* = 0.74 vs. control).

**Conclusion:**

Ever use of oral estrogen was associated with increased SBP and DBP compared to non-oral estrogen use and no use. Given the cardiovascular risk associated with both menopause and increased blood pressure, further studies are required exploring the potential benefits of non-oral estrogen in postmenopausal women.

## Introduction

The population of postmenopausal women will reach over 1 billion globally by the year 2025 (1). The hallmark of the menopausal transition is the presence of vasomotor symptoms such as hot flashes and/or night sweats, which are experienced by over 75% of women and may persist for over 7 years (2). Hormone therapy (HT) remains the most effective treatment for menopausal symptoms, but the decision to use HT is complex and requires individualized balancing of the risks and benefits. Previous studies have indicated an increase in adverse cardiovascular events with HT use (3). Data suggests that the route of estrogen administration may play a role in mediating the effects of exogenous estrogen on cardiovascular risk (4–11), with non-oral routes being associated with lower measures of blood pressure and other cardiovascular risk factors (5, 7, 12, 13).

Previous studies have examined the impact of different routes of estrogen administration on vascular measures and have shown conflicting results (6, 8, 14–19), which is most likely due to differences in study populations, age and cause of menopause, timing of postmenopausal hormone initiation, and notably, the presence of progestin. The Women's Health Initiative (WHI), a trial in which postmenopausal women with a uterus were randomized to both oral conjugated equine estrogen (CEE) and medroxyprogesterone acetate (MPA) or placebo was stopped early due to negative cardiovascular outcomes in the intervention arm (3). However, the WHI trial in which postmenopausal women without a uterus were randomized to only oral CEE without concomitant progestin compared to placebo was conducted to completion and showed no difference in cardiovascular outcomes (20), suggesting that MPA, and progestins in general, play a role independent of estrogen on cardiovascular health (21). The objective of this study was to investigate the association between the route of administration of estrogen exposure, in the absence of concomitant progestin use, on blood pressure and arterial stiffness in postmenopausal women.

## Methods

### Study Design

Strengthening the Reporting of Observational Studies in Epidemiology (STROBE) guidelines for the cross-sectional studies were followed to develop this manuscript (22). This study was a cross-sectional, secondary analysis of the Women's Advanced Risk-assessment in Manitoba (WARM) Hearts Study (Clinical Trial: NCT03938155) (23). The initial study was a prospective cohort study, designed to investigate novel cardiovascular prognostic tools to identify women at elevated risk for experiencing an adverse cardiovascular event over a 5-year period (23). The WARM Hearts Study was approved by the University of Manitoba Health Research Ethics Board and St. Boniface Hospital Research Review Committee (HS22576, H2019:063). The secondary analysis was approved by the Conjoint Health Research Ethics Board of the University of Calgary (REB20-1456). Written informed consent was obtained from all study participants in accordance with the Declaration of Helsinki.

### Participants

The initial observational cohort study was conducted at the St. Boniface Hospital Asper Clinical Research Institute and University of Manitoba Active Living Centre in Winnipeg, Manitoba, Canada. Participants were recruited using a convenience sampling approach through word of mouth, newspaper articles, an online webpage, media interviews, poster advertisements or presentations at community events. Females aged 55 years and older with a Manitoba Personal Health Information Number (PHIN) were included in the study. Participants were excluded if they had previously been hospitalized for the following cardiovascular conditions: ischemic heart disease, acute myocardial infarction, stroke/transient ischemic attack, percutaneous coronary intervention, coronary artery bypass surgery, congestive heart failure, peripheral artery disease, congenital heart disease and arrhythmias. Participants were also excluded if they had received medical advice against participating in physical activity.

All participants underwent a medical history, physical examination, and laboratory screening. All demographic information (e.g., age, sex assigned at birth, current gender identity, race/ethnicity) was determined by self-report. Data on menopausal status (e.g., age at menopause, vasomotor symptoms), previous contraceptive use and reproductive history were obtained using a standardized questionnaire.

### Hormone Therapy Exposure

Participants were categorized based on self-reported current or past HT use (ever use) or controls (no HT use). HT use was categorized based on formulation: (1) estrogen-only; (2) progestin only; (3) combined estrogen and progestin. For the purposes of this study, data on estrogen-only HT use was analyzed to investigate the effect of estrogen, independent of concomitant progestin use, on surrogate cardiovascular markers. Estrogen HT was stratified by route of administration, including oral, transdermal (transdermal patch and gel) and vaginal use.

### Outcomes

The primary outcomes of this exploratory study were the difference in SBP and DBP between (1) oral and non-oral ever users; (2) oral ever users and controls; (3) non-oral ever users and controls; and (4) transdermal and vaginal ever users. The secondary outcomes were the differences in aortic pulse wave velocity (aPWV) and augmentation index (AIx) standardized to 75 beats per minute between (1) oral and non-oral ever users; (2) oral ever users and controls; (3) non-oral ever users and controls; and (4) transdermal and vaginal ever users. aPWV and AIx are measures of arterial stiffness and are validated measures of cardiovascular risk (24). Seated blood pressure measurements were taken at the start of the study visit by placing a standard BP cuff on the left upper arm as per guidelines (25) using the validated Mobil-O-Graph (IEM, Stolberg, Germany). The Mobil-O-Graph is an ambulatory automated BP monitor validated for the non-invasive assessment of brachial blood pressure (26, 27) and estimates of aPWV (28, 29), and AIx simultaneously indirectly using a mathematical transformation of the brachial cuff waveform (30). The device has been validated in healthy individuals and patients with essential hypertension (30, 31), and estimates of aPWV has an acceptable accuracy similar to intra-aortic readings (28) and cardiac magnetic resonance imaging (29). Measurements were taken in triplicate and the second and third measurements were averaged.

### Study Protocol

Participants were studied in the fasting state. Blood was drawn and immediately centrifuged at 2,000 × g for 10 min after collection. Plasma was then aliquoted into 500 μL samples and stored at −80°C. The plasma total cholesterol, high-density lipoprotein (HDL) cholesterol, low-density lipoprotein (LDL) cholesterol, triglycerides, and fasting blood glucose levels of the participants were measured enzymatically.

A pre-exercise BP and heart rate clearance was conducted for participant safety (32). Participants whose measurements exceeded a SBP of 160 mmHg, a diastolic BP of 90 mm Hg or a heart rate of 100 beats per minute were given 5 min to rest before the measurement was retaken. If any of the participant's measurements remained above these values for two consecutive measures, the study day was terminated, and participants were advised to visit their primary healthcare provider for assessment.

### Statistical Analysis

General characteristics of the study population by route of estrogen HT use were presented as the mean ± SE or median and interquartile range (IQR) as appropriate for continuous variables and proportions for dichotomous variables. Separate analyses of current estrogen-only use participants and former estrogen-only use were conducted. Differences in baseline characteristics across categories for HT were compared using a one-way ANOVA. Bonferroni *post-hoc* test was performed to adjust for multiple comparisons and identify significant differences between groups. Associations were evaluated using linear regression analysis and presented with the beta coefficient and 95% confidence intervals (CI). A forward stepwise multivariate regression analysis was used to assess the contribution of covariates. Models were adjusted based on factors known to be associated with outcome and exposure. The primary outcome (SBP and DBP) was adjusted for age and body mass index. aPWV was adjusted for age, mean arterial pressure, age at menopausal onset, history of vasomotor symptoms, and history of hypertension. As the augmentation index is dependent on age (33), sex (33), and heart rate (34), no additional adjustments were made for any of the above-mentioned variables for the following reasons: (1) the calculation for AIx already adjusts for age; (2) the study population was entirely female; and (3) AIx is standardized to 75 beats per minute. The AIx was adjusted for age at menopausal onset (35, 36), history of vasomotor symptoms (37), and history of hypertension (38). Due to significant collinearity among hypertension, diabetes, chronic kidney disease, and myocardial infarction identified by increased variance inflation factors, only hypertension was included as a covariate in the model. Separate analyses were conducted with either controls or oral estrogen ever use as the referent group. Assumptions for both linear and logistic regression models were tested. Sensitivity analyses were conducted by stratifying participants by former or current estrogen-only HT use status. All analyses were conducted using STATA (version 9.13; College Station, TX, USA) with a two-tailed significance level of <0.05.

## Results

### Study Cohort

**Figure 1:**
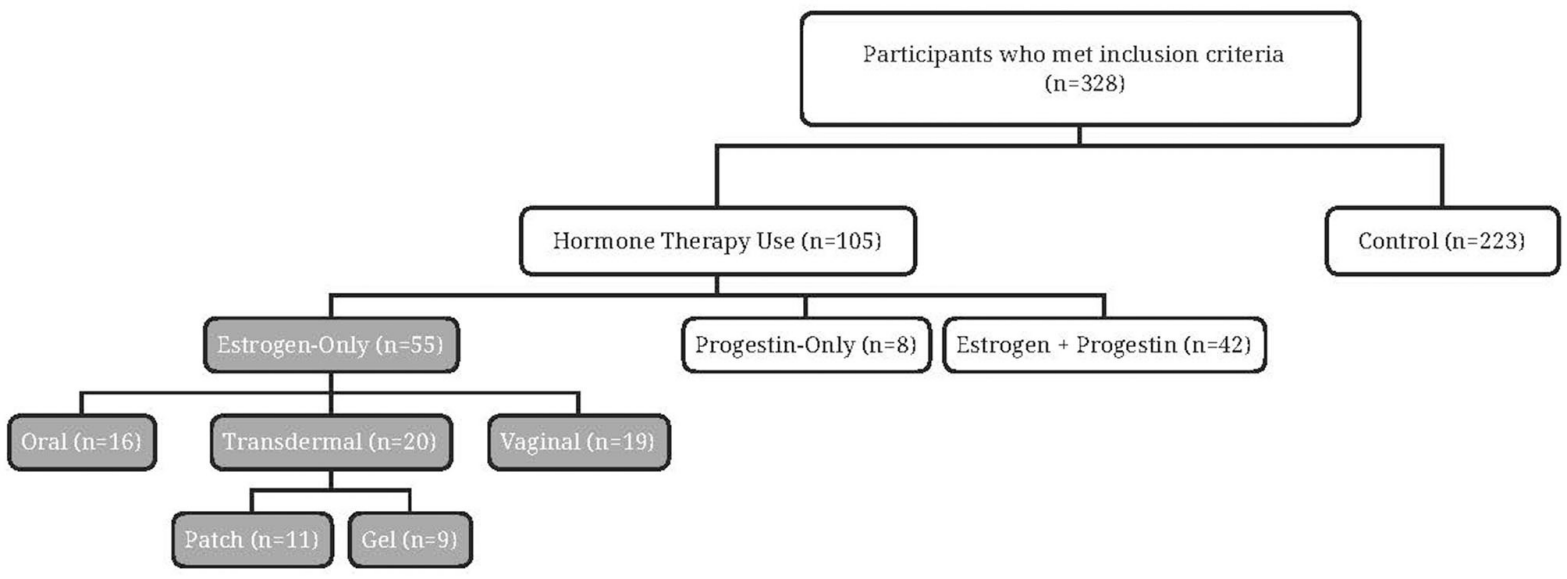
Flow diagram of study participants.

Of the 328 participants recruited (Figure 1), the majority (*n* = 223) reported never using HT. Approximately one-third (*n* = 105) reported current or past use of HT and just over half reported estrogen-only HT (*n* = 55). Among the oral estrogen-only HT users, few participants were currently using oral estrogen (*n* = 2), with most reporting past use (*n* = 14). Similar numbers of current (*n* = 20) and past estrogen use (*n* = 19) were reported by non-oral (transdermal and vaginal) estrogen-only HT users.

### Baseline Characteristics

All participants reported being assigned female sex at birth and self-identified as women. The majority of participants self-reported as white (96%) and were between 60 and 69 years of age (Table 1). Participants who reported use of oral estrogen were older than non-oral estrogen users and controls (*p* < 0.001 and *p* = 0.01, respectively). Duration of estrogen use was similar across the groups (median, [IQR]. Oral: 4.5 [2, 9] years; transdermal: 4.5 [0, 9] years; vaginal: 4.5 [1, 9] years). Oral users reported reaching menopause at a younger age compared to controls (*p* < 0.001); however, there was no difference in age of menopausal onset between oral and non-oral users (*p* = 0.8). Most oral estrogen HT users reported surgical menopause, while all non-oral estrogen HT users and control achieved menopause naturally. The majority of non-oral users of estrogen had a previous history of tobacco use.

**Table 1:** Baseline characteristics.

		**HT Users (*****n*** **=** **55)**		
	**Control (*n* = 223)**	**Oral** **(*n* = 16)**	**Transdermal** **(*n* = 20)**	**Vaginal** **(*n* = 19)**
Age y, mean (SE)	63 (0.37)^a^	68 (1.5)^b^	61 (1.2)^a^	62 (1.1)^a^
BMI (kg/m^2^)	26.3 (1)	27 (0.3)	27 (0.9)	26 (0.7)
Heart rate (bpm), mean (SE)	68 (0.5)	71 (2)	66 (2)^a^	69 (2)
Age groups, *n* (%)				
55–59	65 (30)	-	11 (55)	6 (31)
60–69	127 (57)	10 (63)	7 (35)	10 (53)
70–79	28 (12)	6 (37)	2 (10)	2 (11)
>80	3 (1)	-	-	1 (5)
Race (% White)	214 (96)	16 (100)	20 (100)	19 (100)
Education, *n* (%)				
No degree	17 (8)	1 (6)	1 (5)	1 (5)
Highschool	35 (15)	2 (13)	1 (5)	-
Post-secondary	171 (77)	13 (81)	18 (90)	18 (95)
Marital status (*n*, % married)	172 (77)	8 (50)	18 (90)	15 (79)
Age of menopause onset, mean (SE)	51 (0.3)	46 (3)^b^	49 (1.5)	47(2)
Menopause status, *n* (%)				
Natural	188 (84)	6 (38)	9 (45)	15 (79)
Surgical	7 (3)	8 (50)	6 (3)	-
Chemotherapy	2 (1)	-	-	-
Other	25 (11)	2 (12)	5 (25)	4 (21)
Vasomotor symptoms, *n* (%)		11 (69)	16 (80)	14 (74)
Hormone therapy use, *n*				
Current	-	2	10	10
Past	-	14	10	9
Duration of use, y (median [IQR])	-	4.5 [2, 9]	4.5 [0, 9]	4.5 [1, 9]
Previous contraceptive use, *n* (%)	191 (86)	10 (63)	19 (95)	19 (100)
Oral contraceptive	188 (84)	9 (56)	18 (90)	19 (100)
IUD	34 (15)	3 (19)	-	7 (37)
Ring	4 (2)	-	-	1 (5)
Injectable	2 (1)	-	-	-
Pregnancy, *n* (%)	178 (80)	12 (75)	17 (85)	17 (90)
Self-reported medical conditions, *n* (%)				
Hypertension	52 (23)	4 (25)	3 (15)	0
Diabetes	4 (2)	1 (6)	-	-
Chronic kidney disease	1 (1)	1 (6)	-	-
PCOS	5 (2)	-	1 (5)	1 (5)
Cancer	32 (14)	3 (19)	3 (15)	2 (10)
Pre-eclampsia	10 (4)	-	-	1 (5)
Previous tobacco use	96 (43)	8 (50)	13 (80)	11 (58)
Current tobacco use	3 (1)	1 (6)	1 (5)	-
Lipid values, mean (SE)				
HDL (mmol/L)	1.6 (0.03)	1.6 (0.7)	1.8 (0.1)^a^, ^b^	1.8 (0.9)^a^, ^b^
LDL (mmol/L)	3.4 (0.06)	3.7 (0.2)	3.2 (0.2)^a^	3.5 (0.2)
Total cholesterol (mmol/L)	5.2 (0.06)	5.5 (0.2)	5.4(0.2)	5.2 (0.2)
Triglycerides (mmol/L)	1.11 (0.03)	1.4 (0.13)	0.91 (0.07)^a^	1.24 (19)

*Data are represented as mean (SE), No. (%), or median [IQR]. y, years; IUD, intra-uterine device; PCOS, polycystic ovarian syndrome; HT, hormone therapy; HDL, high density lipoprotein; LDL, low density lipoprotein.*

a *p <0.05 compared to oral HT users.*

b *p <0.05 compared to controls*.

### Blood Pressure

**Figure 2:**
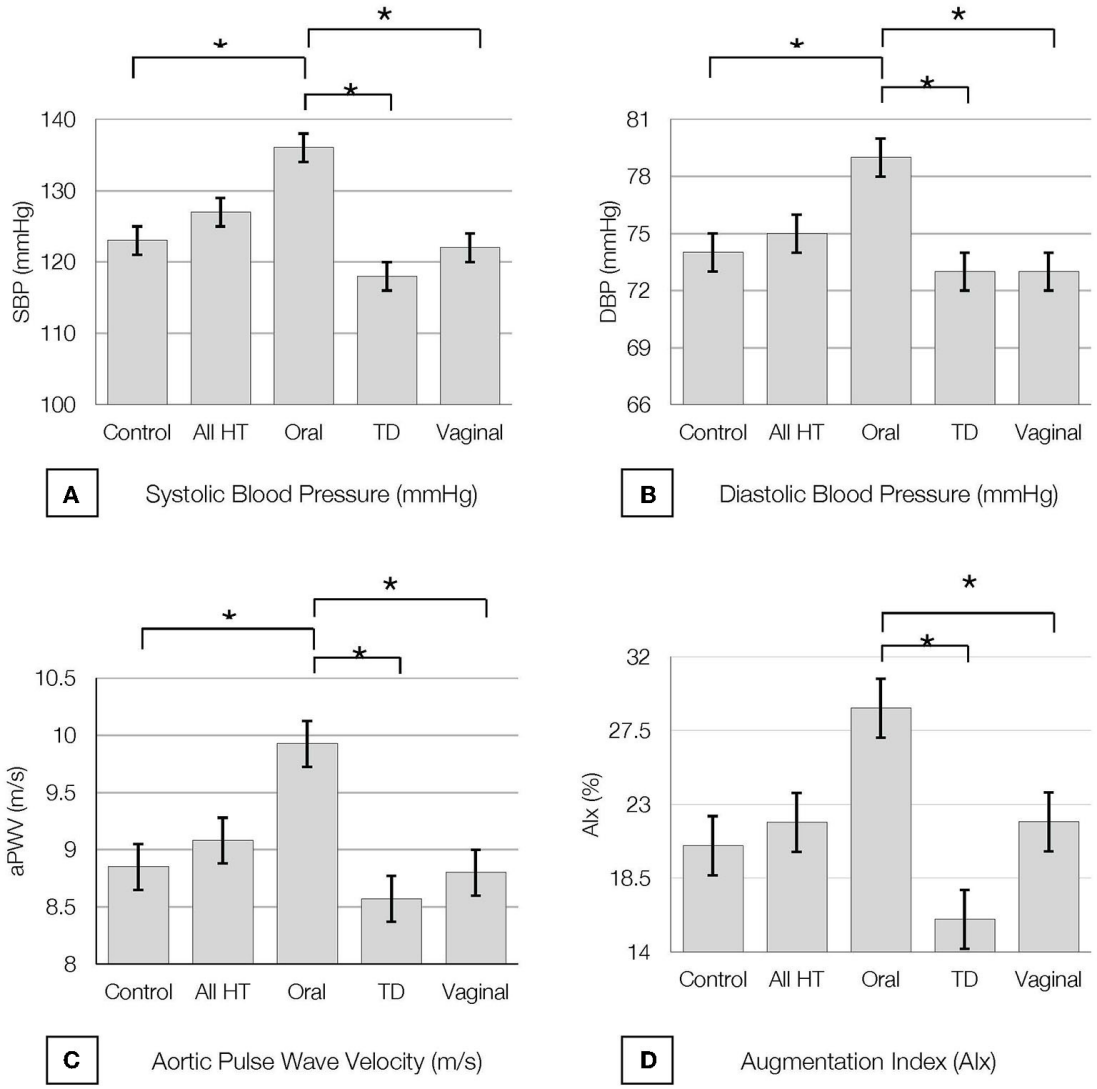
Baseline **(A)** Systolic Blood Pressure (SBP), **(B)** Diastolic Blood Pressure (DBP), **(C)** Pulse Wave Velocity (PWV) and **(D)** Augmentation Index at 75 bpm (AI@75) by hormone therapy type. All values are presented as mean ± SE.* indicates *P* < 0.05. All HT, all hormone therapy users; TD, transdermal.

All study participants demonstrated BP readings within the normotensive range and there were no differences in BP measures between estrogen users and controls (*p* = 0.8). However, SBP and DBP were increased in oral estrogen users compared to non-oral estrogen users (transdermal: SBP *p* <0.01; DBP, *p* = 0.012; vaginal: SBP *p* = 0.02; DBP, *p* = 0.01) and controls (SBP *p* = 0.03; DBP, *p* = 0.02; Table 2 and Figure 2). These associations remained significant after adjustment for age and BMI (Tables 3, 4). No differences in SBP or DBP were observed between transdermal and vaginal estrogen users (*p* = 0.79), or transdermal and vaginal estrogen users and controls (*p* = 0.83). Separate sensitivity analyses of current estrogen-only use participants and former estrogen-only use participants did not show any differences in SBP or DBP across different routes of estrogen (Tables 3, 4).

**Table 2:** Baseline hemodynamic and arterial stiffness measures.

		**HT users (*****n*** **=** **55)**		
	**Control** **(*n* = 223)**	**Oral** **(*n* = 16)**	**Transdermal (*n* = 20)**	**Vaginal (*n* = 19)**
SBP (mmHg)	124 (1)^a^	137 (4)^b^	118.2 (2)^a^	122.8 (2)^a^
DBP (mmHg)	74 (1)^a^	79 (2)^b^	73 (1)^a^	73 (2)^a^
aPWV (m/s)	8.85 (1)^a^	9.93 (1)^b^	8.57 (1)^a^	8.80 (1)^a^
AIx (%)	20.5(4)	28.9 (2)	16.0 (2)^a^	21.9 (2)^a^

*AIx, Augmentation Index at 75 bpm; DBP, diastolic blood pressure; HT, hormone therapy; aPWV, aortic pulse wave velocity; SBP, systolic blood pressure.*

a *p <0.05 compared to oral HT users.*

b *p <0.05 compared to controls*.

**Table 3:** Multi-variate analysis of SBP by route of administration of hormone therapy type presented as beta coefficients, [95% CI].

	**Ever use**	**Current use**	**Past use**
**Control (reference group)**			
Oral	9.4 [3, 16]^a^	-	5.4 [−2, 12]
Transdermal	−4.5 [−10, 1]	−8.9 [−24, 6]	−0.3 [−8, 7]
Vaginal	−0.7 [−7, 5]	−13 [−32, 4]	−0.9 [−9, 8]
**Oral (reference group)**			
Transdermal	−17 [−32, −3]^a^	-	−0.3 [−1, 0.3]
Vaginal	−13.6 [−25, −2]^a^	-	−0.4 [−1, 0.4]

*Only two participants were currently using oral HT, therefore not enough participants to run analyses.*

*Adjusted for age, BMI.*

a *p <0.05 compared to reference group*.

**Table 4:** Multi-variate analysis of DBP by route of administration of hormone therapy type presented as beta coefficients, [95% CI].

	**Ever use**	**Current use**	**Past use**
**Control (reference group)**			
Oral	5.4 [0.8, 10]^a^	-	3.8 [−1, 8]
Transdermal	−0.5 [−5, 3]	0.04 [−0.4, 0.3]	1.9 [−4, 8]
Vaginal	−0.03 [−0.3, 0.3]	−0.05 [−0.4, 0.3]	−1.4 [−7, 5]
**Oral (reference group)**			
Transdermal	−11 [−19, −3]^a^	-	−6.3 [−15, 2.]
Vaginal	−6.7 [−14, −0.2]^a^	-	−9.4 [−19, 1]

*Only two participants were currently using oral HT, therefore not enough participants to run analyses.*

*Adjusted for age, BMI.*

a *p <0.05 compared to reference group*.

### Pulse Wave Velocity

All study participants had aPWV measurements in the normal range (24, 39) and there were no differences in aPWV measures between estrogen users and controls (*p* = 0.7). Oral estrogen users demonstrated increased aPWV compared to both transdermal (*p* < 0.01) and vaginal (*p* = 0.03) estrogen users as well as controls (*p* = 0.03) (Table 2 and Figure 2), but after adjustment for covariates, route of estrogen delivery was not associated with aPWV (Supplementary Table 1). No differences were observed within non-oral estrogen user groups or between non-oral (transdermal and vaginal) estrogen users and controls. Separate sensitivity analyses of current estrogen-only use participants and former estrogen-only use participants did not show any differences in aPWV across different routes of estrogen delivery (Supplementary Table 1).

### Augmentation Index

All study participants had AIx measurements in the normal range (39) and there were no differences in AIx measures between estrogen users and controls (*p* = 0.6). Oral estrogen users demonstrated increased AIx compared to non-oral estrogen users (transdermal: *p* < 0.01; vaginal: *p* = 0.04) (Table 2 and Figure 2), but after adjustment for covariates, route of estrogen delivery was not associated with AIx (Supplementary Table 2). No differences were observed between transdermal and vaginal users, or non-oral estrogen users and controls. Separate sensitivity analyses of current estrogen-only use participants and former estrogen-only use participants did not show any differences in AIx across different routes of estrogen delivery (Supplementary Table 2).

## Discussion

To the best of our knowledge, this is the largest observational study examining the association between the route of administration of estrogen HT in the absence of a concomitant progestin on measures of BP and arterial stiffness in postmenopausal women. The key findings of this study are as follows: (1) ever use of oral estrogen was associated with increased SBP and DBP compared to ever use of non-oral estrogen (transdermal and vaginal) and never use (control), even after adjustment for covariates; (2) ever use of non-oral estrogen was associated with similar SBP and DBP compared to never use (control); (3) ever use of transdermal and vaginal estrogen was associated with similar measures of SBP and DBP; (4) arterial stiffness measures (aPWV and AIx) were similar between oral, non-oral and never use (control). The data suggests that oral estrogen use alone, without concomitant progestin, is associated with increased SBP and DBP compared to non-oral estrogen use and controls. Putting the difference in SBP into clinical context, compared with either non-oral estrogen or never use of estrogen, the 30 year risk of cardiovascular disease associated with this increase in blood pressure amongst oral estrogen users is almost 30% higher (40) - roughly the remaining lifespan after menopause (41).

Oral estrogens undergo hepatic first-pass metabolism, which has been associated with activation of the renin-angiotensin-aldosterone system (RAAS) and increased levels of circulating angiotensin II (6, 7, 42, 43). In contrast, non-oral delivery methods, such as transdermal, injectable, or transvaginal estrogen bypass the liver and are not associated with upregulated RAAS activity. Through bypass of the digestive tract and liver, transdermal estrogen formulations have the advantage of delivering unmetabolized estradiol directly to the bloodstream and thus require lower doses compared with oral agents. The Women's Health Initiative Observational Study reported that the odds of incident treated hypertension after 3 years did not vary according to dose of estrogen (44), and estradiol levels have not been shown to be associated with cardiovascular risk (45), at least in users of oral estrogen, suggesting different metabolic pathways likely play a role in the different risk profiles seen between oral and non-oral routes of estrogen administration (46).

Blood pressure is a major modifiable risk factor for cardiovascular disease (47). Blood pressure often increases in the menopausal transition, and while it has been suggested that this is due to decreases in estrogen levels (48), this has not been definitively established (49, 50). Studies on the effect of postmenopausal estrogen therapy on blood pressure are conflicting. The Heart and Estrogen/progestin Replacement Study (HERS), a randomized placebo-controlled trial of 2,763 postmenopausal women with a history of cardiovascular disease, showed a 2 mmHg increase in mean SBP with oral CEE + MPA use, although this was not the primary outcome (14). Similarly, the WHI examined the effect of these same interventions on primary prevention of cardiovascular disease and reported that in postmenopausal women, oral CEE and CEE+MPA at conventional doses both increased mean SBP compared to placebo, an effect that was most pronounced in younger women and those who were Hispanic or White (15). In contrast, a 3-year, multicenter, randomized, double-blind, placebo-controlled trial of 875 healthy postmenopausal women aged 45 to 64 years showed that CEE alone or in combination with progestin had no effect on blood pressure (16). Similarly, a four-year randomized trial of 727 younger postmenopausal women randomized to either transdermal estradiol or oral CEE (with both groups taking cyclical micronized progesterone) showed similar changes in BP (8). Of note, in addition to the use of progesterone, the participants in this study were recently menopausal and on average more than a decade younger than those in our study population, which may account for the lack of differences in blood pressure with the use of different routes of estrogen administration.

Previous work has shown differential changes in blood pressure with oral and non-oral routes of estrogen administration. In a randomized trial of postmenopausal women, 28 participants received continuous oral CEE plus cyclic MPA, 28 received a continuous transdermal estradiol patch plus cyclic MPA, and 27 did not receive either therapy (51). After 12 months, there were no differences in BP across groups, although the transdermal group demonstrated a decrease in arterial stiffness. Our study population differs from these in that participants were taking estrogen-only hormone therapy to exclude any potential effects of progestins on the outcomes of interest. A non-randomized, prospective study of 90 normotensive, oophorectomized women, aged 30–59 years on either oral (*n* = 50) or transdermal (*n* = 40) estrogen therapy demonstrated a decrease in blood pressure after 6 months in the transdermal group, with no change in the oral group (17). However, there was significant variability in individual responses with BP increasing in more than one-third of the women on either treatment, in keeping with recent findings from the Study of Women's Health Across the Nation (SWAN) cohort demonstrating distinct BP trajectories over the menopause transition that were independent of estradiol levels or hormone therapy use (49). A randomized trial of 38 younger (averages ages 54.6 and 55.5 years) normotensive women with natural menopause compared the effects of transdermal estradiol and oral CEE and MPA (6), and reported a decrease in DBP (−3 mmHg) and mean BP (−3.2 mmHg) after 12 months in the transdermal group, whereas the oral group did not demonstrate any changes in BP. A randomized crossover placebo-controlled study in 12 normotensive postmenopausal women (53 ± 2 years of age, 10 ± 3 years after the last menstrual period) examined the effects of 8 weeks of transdermal estradiol (200 microgram/d), oral conjugated estrogens (0.625 mg/d), or placebo (52). After 8 weeks of transdermal estrogen, ambulatory diastolic BP fell by 5 ± 2 mm Hg (*p* = 0.0003) but no change was observed with oral CEE or placebo. The reported discrepancies across studies of the effect of route of estrogen administration on blood pressure likely reflect differences in estrogen dose, formulation, populations, age, cause, and age at menopause as well as timing of initiation of hormone therapy. Many of these studies examined the effects of combined estrogen and progestins, with may have also impacted outcomes. A strength of the present study is the inclusion of participants with no concomitant progestin use.

Arterial stiffness is a validated predictor of cardiovascular morbidity and mortality (24, 53). There is limited data on the effect of postmenopausal hormone therapy on arterial stiffness. A study of 52 postmenopausal women demonstrated greater arterial compliance in hormone therapy users compared to non-users, and greater arterial compliance in estrogen-only users compared to combined estrogen and progestin-users (18). Interestingly, arterial compliance increased significantly in hormone users after 4 weeks of cessation, but the authors did not report the route of estrogen administration or concomitant progestin use (18). Conversely, a recent cross-sectional study (19) of 36 menopausal women reported that hormone therapy (six oral, two transdermal estrogen)-users had higher aPWV, consistent with greater cardiovascular risk, than postmenopausal non-users (*n* = 26), although the authors did not report whether hormone therapy included a progestin. Similar to studies examining the effect of postmenopausal hormone therapy on blood pressure, it is possible that factors other than the route of estrogen administration contributed to the reported outcomes, including differing methodologies to measure arterial stiffness (54).

This study has strengths and limitations. First, because of the self-reported nature of HT exposure in this study, recall bias leading to misclassification of hormone exposure is a possibility. However, most studies comparing self-reports of HT with physician/pharmacy records have shown moderate to good agreement (55). Next, most oral estrogen users in this study underwent surgical menopause while in contrast, non-oral estrogen users and controls underwent natural menopause. Recall of age at menopause has been shown to be better for surgical menopause than for natural menopause (56, 57). Because estrogen therapy is often started around menopause, it is reasonable to assume that recall of age at first use and duration would be similarly affected. Overall, the agreement between self-report and the medical record on the use of oral estrogens is at least moderate (58). Since participants using oral estrogen in our study were more likely to undergo surgical menopause, this suggests that they were more likely to initiate estrogen at the time of menopause, which is associated with improved cardiovascular outcomes (59). It is important to note that the type of estrogen used was not reported. The most commonly used oral estrogen formulations, at least in the United States, for systemic treatment of menopausal symptoms are estradiol and conjugated equine estrogens (CEE) (60), whereas transdermal and vaginal forms of estrogen are exclusively estradiol (61); estradiol exposure is associated with better cardiovascular outcomes compared to CEE (62) and it is possible that type of estrogen also played a role in our outcomes of interest. Non-oral HT users were younger and prescribed fewer cardiovascular medications than their oral estrogen counterparts (63). As such, we included age and medical comorbidities in our linear regression analyses to mitigate these potential differences. Of note, history of tobacco use was higher in the non-oral estrogen group; it is thus possible that the risk demonstrated with oral estrogen use in our study may actually be an underestimate. Lastly, the study population was restricted to ever users of estrogen-only menopausal HT with the majority being past, rather than current, users. The results may thus not be applicable to women who use combined estrogen and progestin HT or who currently use estrogen only HT. However, an important strength of this study is that limiting our study population to estrogen-only users, we were able to investigate the independent associations of route of administration estrogen exposure on measures of blood pressure and arterial stiffness while minimizing confounding factors.

In this community-dwelling postmenopausal population, oral estrogen HT use, without concomitant progestin, was associated with increased SBP and DBP. Menopause is associated with increased cardiovascular risk (64). Vasomotor symptoms (65) are common and the guideline-recommended treatment is estrogen with or without concomitant progestin (66). Therefore, an understanding of the potential cardiovascular benefits and risks of different routes of administration of estrogen HT will allow individuals and their health care providers to make informed decisions regarding therapy during this important transition time. Although it is unclear if the effects of different routes of administration of estrogen use on blood pressure in the absence of a progestin are limited to a particular time period, or whether the timing of initiation or duration of use plays a role, the association between oral estrogen use and increased blood pressure in our study warrants attention in the growing postmenopausal population.

## Data Availability Statement

The original contributions presented in the study are included in the article/Supplementary Material, further inquiries can be directed to the corresponding author/s.

## Ethics Statement

The studies involving human participants were reviewed and approved by Conjoint Health Research Ethics Board of the University of Calgary (REB20-1456) and University of Manitoba Health Research Ethics Board and St. Boniface Hospital Research Review Committee (HS22576, H2019:063). The patients/participants provided their written informed consent to participate in this study.

## Author Contributions

CK and SA prepared the concept, designed the study, performed statistical analysis, and prepared the manuscript. JH, KB, and TD recruited participants and collected the data. All authors were involved in the interpretation of the results and the revision of the manuscript and approved the submitted version of the manuscript.

## Funding

CK was funded by the Libin Cardiovascular Institute Doctoral Scholarship in Women's Cardiovascular Health and the Canadian Institute of Health Research-Leaders in Medicine MD/PhD award. JH was funded by the Pawan K. Singal Graduate Scholarship in Cardiovascular Sciences and Kappa Kappa Gamma Foundation of Canada Scholarship. KB was funded by a Frederick Banting & Charles Best Canada Graduate Scholarships – Doctoral Award. TD holds a St. Boniface Hospital Research Foundation Molson Women's Heart Health Research grant to conduct the WARM Hearts study.

## Conflict of Interest

The authors declare that the research was conducted in the absence of any commercial or financial relationships that could be construed as a potential conflict of interest.

## Publisher's Note

All claims expressed in this article are solely those of the authors and do not necessarily represent those of their affiliated organizations, or those of the publisher, the editors and the reviewers. Any product that may be evaluated in this article, or claim that may be made by its manufacturer, is not guaranteed or endorsed by the publisher.
