# Supplementary material for: The Association Between Route of Post-menopausal Estrogen Administration and Blood Pressure and Arterial Stiffness in Community-Dwelling Women
Source: Front Cardiovasc Med. 2022 Jun 10;9:913609. doi: 10.3389/fcvm.2022.913609 (PMC9226418; doi:10.3389/fcvm.2022.913609)
Supplement: Supplementary file 1 [file Table_1.pdf]

Supplementary Tables

Table 1: Multi-variate analysis of aPWV by route of administration of Hormone therapy presented as beta coefficients, [95% CI]

	Ever Use	Current Use	Past Use
Control (Reference Group)			
Oral	0.03 [-0.2 , 0.3]	-	0.13 [-0.2 , 0.5]
Transdermal	-0.05 [-0.3 , 0.2]	0.04 [-0.4 , 0.3]	-0.05 [-0.4 , 0.3]
Vaginal	-0.03 [-0.3 , 0.30	-0.05 [-0.4 , 0.3]	-0.04 [-0.4 , 0.3]
Oral (Reference Group)			
Transdermal	-0.4 [-0.8 , 0.1]	-	-0.3 [-1 , 0.3]
Vaginal	-0.4 [-1 , 0.2]	-	-0.4 [-1 , 0.4]

Only 2 participants were currently using oral HT, therefore not enough participants to run analyses.
Adjusted for age, mean arterial pressure, hypertension, age of menopausal onset, and vasomotor symptoms.
[a] p<0.05 compared to reference group
