# Supplementary material for: The Association Between Route of Post-menopausal Estrogen Administration and Blood Pressure and Arterial Stiffness in Community-Dwelling Women
Source: Front Cardiovasc Med. 2022 Jun 10;9:913609. doi: 10.3389/fcvm.2022.913609 (PMC9226418; doi:10.3389/fcvm.2022.913609)
Supplement: Supplementary file 2 [file Table_2.pdf]

Supplementary Tables

Table 2: Multi-variate analysis of AIx by route of administration of hormone therapy presented as beta coefficients, [95% CI]

	Ever Use	Current Use	Past Use
Control (Reference Group)			
Oral	1.2 [-6 , 9]	-	0.2 [-8 , 8]
Transdermal	-5.2 [-11, 0.6]	-1.8 [-9 , 6].	-1.80 [-9 , 6]
Vaginal	1.7 [-4, 8]	2.4 [-6 , 11]	2.4 [-6 , 11]
Oral (Reference Group)			
Transdermal	-5.3 [-0.8 , 0.1]	-	3 [-15 , 21]
Vaginal	-2 [-18 , 14]	-	-2.3 [-21 , 26]

Only 2 participants were currently using oral HT, therefore not enough participants to run analyses
Adjusted for hypertension, age of menopausal onset, and vasomotor symptoms.
[a] $p<0.05$ compared to reference group
